# High-pressure polymorphs of ZnCO_3_: Evolutionary crystal structure prediction

**DOI:** 10.1038/srep05172

**Published:** 2014-06-04

**Authors:** A. Bouibes, A. Zaoui

**Affiliations:** 1 LGCgE, Polytech'Lille, University of Lille1. Cite Scientifique, Avenue Paul Langevin, 59655 Villeneuve d'Ascq, France

## Abstract

The high-pressure behavior of zinc carbonate ZnCO_3_ has been investigated using universal structure prediction method together with the density functional theory. In order to explore all possible structures under pressure, separate calculations at high pressure are done here with increasing number of formula units in the unit cell. Two pressures induced phase transitions were considered. The first one occurs at 78 GPa and the second one at 121 GPa. The most stable ZnCO_3_ at ambient condition corresponds to the space group R-3c (phase I), which is in favorable agreement with experiment. The structure with C2/m space group (phase II) becomes stable between 78 GPa and 121 GPa. Finally, the structure with the space group P2_1_2_1_2_1_ (phase III) becomes the most stable when the pressure achieves 121 GPa. Some mechanical properties of R-3c structure were –additionally- calculated and compared with the experimental and previous theoretical data. The resulting behaviors support our findings and confirm the obtained phase transition. Besides, from the analysis of the electronic charge density it comes that at 78 GPa, new bond between oxygen and zinc is formed, what is likely the main cause behind the phase transition.

High-pressure polymorphs of carbon-bearing minerals are important to understand the circulation of carbon in the Earth's interior. Carbonates have been, for a long time, considered as important reservoirs of carbon in deep Earth. High-pressure phases of carbonates are probably among the host minerals for carbon that are present deeply in the mantle. For these reasons, phase transitions and physical properties of high-pressure phases related to carbonates have been subject of intense investigations^1^,^2^,^3^,^4^,^5^. Interesting crystal chemical changes that occur in carbonates under pressure, such as increase of coordination numbers and polymerization of carbonate-ions, may result on new useful materials.

Previous theoretical and experimental studies have focus on common carbonates such as magnesite (MgCO_3_)^6^,^7^,^8^,^9^ and calcite (CaCO_3_)^10^,^11^,^12^,^13^. For instance, it has been proved that the magnesite is stable at pressure up to 80 GPa^6^ and the calcite up to 3.3 GPa^10^. Magnesite goes through two phases transition, from magnesite phase I to magnesite phase II; and from phase II to phase III at 82 and 138 GPa respectively as reported by Oganov et al.^7^. In another work of Oganov et al.^11^, calcite was found to go through three phase transitions at pressures up to 150 GPa from calcite to aragonite at 4 GPa; from aragonite to post-aragonite at 42 GPa, and then to the C222_1_ phase at 137 GPa^7^.

ZnCO_3_ is one of carbon-bearing phases known at the surface at the Earth. This mineral has the same structure type as calcite. However, the knowledge of this zinc carbonate at high pressure remains still very limited. Only few experimental studies of smithsonite have been reported in the literature^14^,^15^. Graf^14^ has presented and defined the structure of ZnCO_3_. Zhang and Reeder^15^ performed experimentally the structural properties of smithsonite and they have also determined the bulk modulus. Recently, Bouibes et al.^16^ determined the ground state properties of smithsonite including structural, mechanical, electronic and bonding properties. Even if there is no clear study indicating the direct importance of zinc carbonate on interior earth, this mineral belongs to group II of carbonates such as MgCO_3_, MnCO_3_, FeCO_3_^17^. Group II contains the most solid minerals needing a high pressure to crystallize. Several studies were performed on FeCO_3_ and MgCO_3_ because of their importance for earth science at high pressure. On the other hand, further study shows that high CO_2_ (g) partial pressure results in zinc carbonate being stable and potentially limit zinc mobility^18^.

The main goal here is to shed light on the structural evolution of ZnCO_3_ at high-pressure. To this end, high-pressure phase transitions will be investigated using the USPEX method/code^19^,^20^. This method has shown great success in numerous applications, including carbonates^19^.

## Results

**Figure 1:**
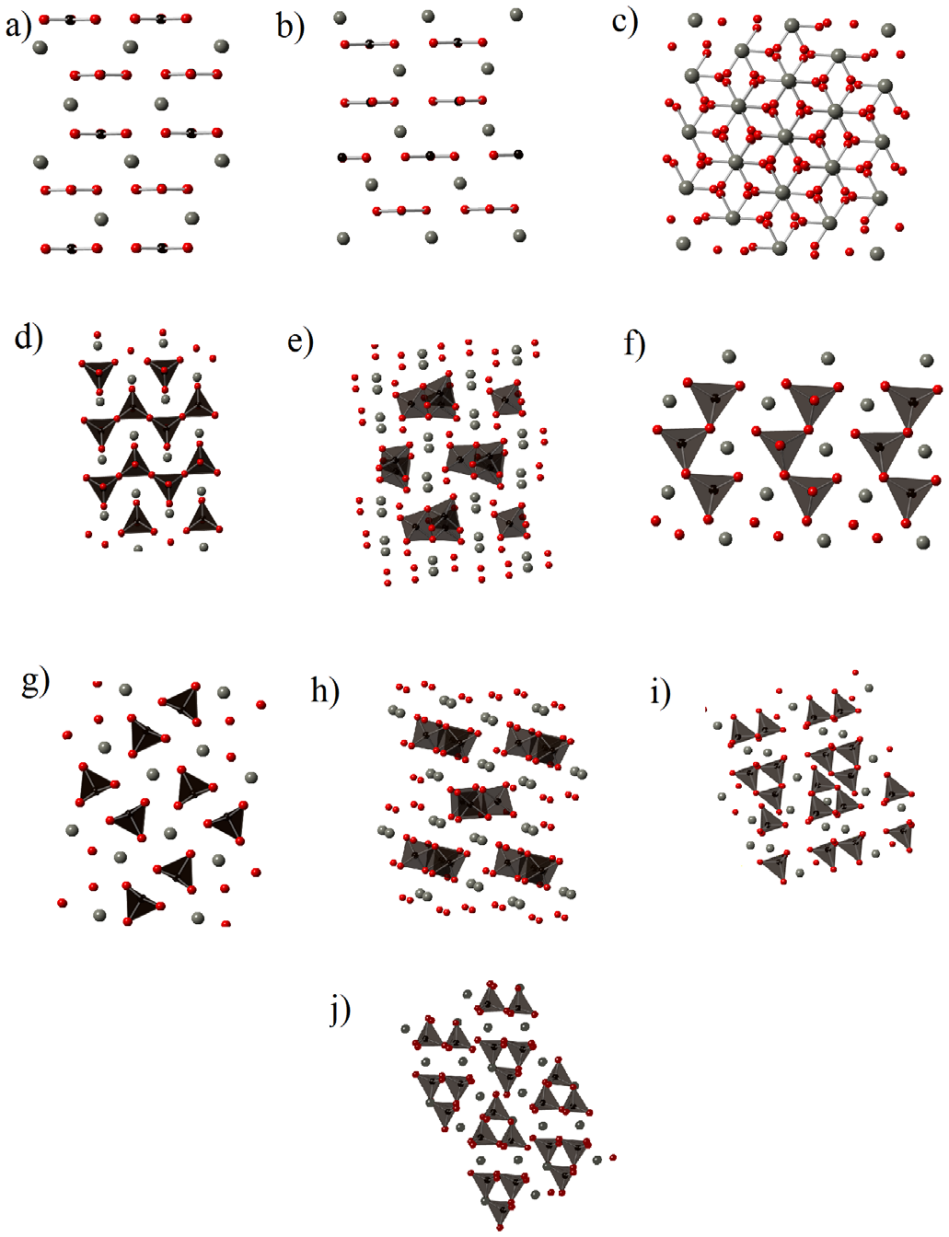
Lowest-enthalpy structures for ZnCO_3_: P-31c (a); P3 (b); R-3c (Phase I) (c); Pbcm (d); P2_1_2_1_2_1_ (Phase III) (e); Pca2_1_ (f); Pnma (g); Pna2_1_ (h); C2/m (Phase II) (i); P2_1_ (j).

In Fig. 1, we display some ZnCO_3_ structures with lowest enthalpy. The obtained lattice parameters as well as bulk modulus and its pressure derivative for the most stable structure (R-3c) are summarized in Table 1.

Three structures corresponding to the space groups R-3c, P-31c and P3, contain triangular (CO_3_)^2−^ ions, which are flat and coplanar. However, two structures with C2/m and P2_1_ space group (30 atoms in the primitive cell), are characterized by a three-membered ring (C_3_O_9_)^6−^ of corner-sharing carbonate tetrahedra. These latter structures were previously found as magnesium carbonate MgCO_3_ stable structure^7^. The structure with C2/m space group corresponds to MgCO_3_ structure that is stable between 82 GPa and 138 GPa and it is called magnesite phase II. In addition, the structure with P2_1_ space group, called magnesite phase III, is the structure of MgCO_3_ above 138 GPa. The structure with space group Pnma is composed of a serial pentaedra carbonate. All the remaining structures contain carbonates (CO_4_)^4−^ tetrahedra.

**Figure 2:**
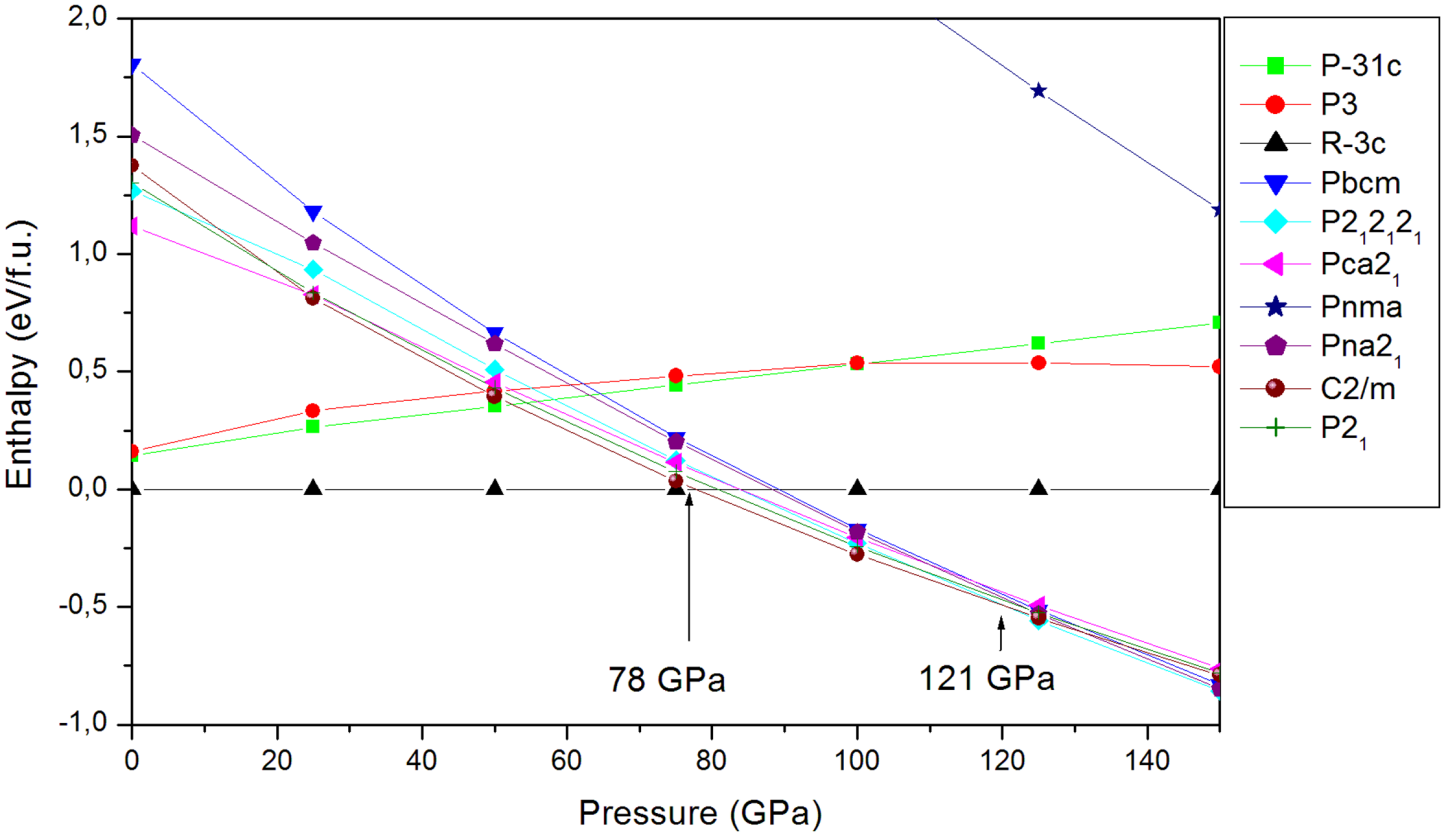
Enthalpies of the best structures vs pressure at 78 GPa (C2/m: Phase II) and at 121 GPa (P2_1_2_1_2_1_: Phase III).

Having determined the most promising structures, we optimized them with very strict computational conditions at pressures ranging from 0 GPa to 150 GPa. Fig. 2 shows the enthalpy as a function of pressure. The most stable structure of ZnCO_3_ at ambient conditions is the R-3c space group structure (calcite structure), which fits perfectly the experiment^15^. The first phase transition occurs at 78 GPa. Under increasing pressure, the second phase transition occurs at 121 GPa. Between 78 GPa and 121 GPa the most stable structure has a space group C2/m with 30 atoms in the primitive cell. This structure is detailed in Table 2. Above 121 GPa, the structure of P2_1_2_1_2_1_ space group becomes more stable, as presented in Table 3.

## Discussion

**Figure 3:**
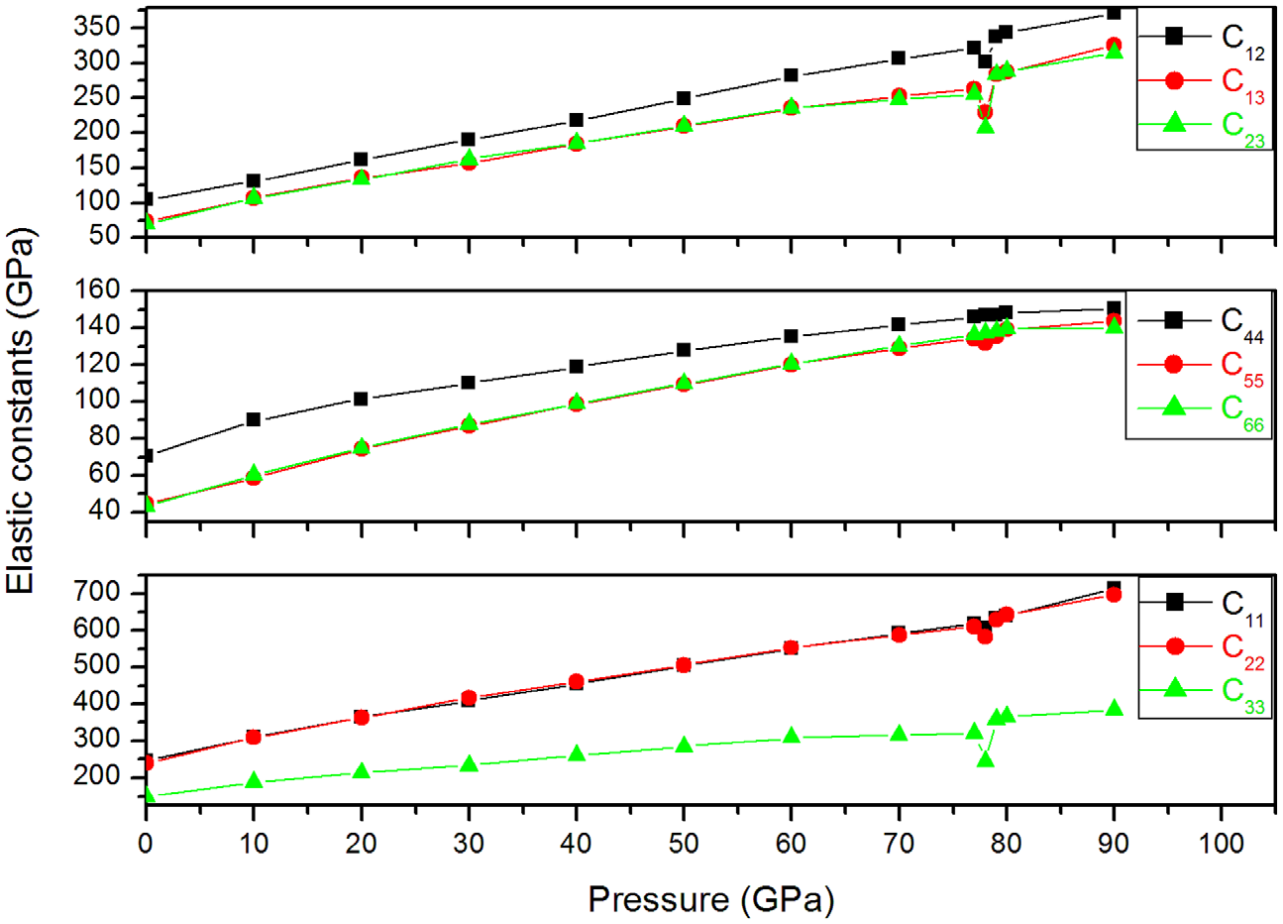
Variation of the elastic constants C_11_, C_22_ and C_33_; C_44_, C_55_ and C_66_; and C_12_, C_13_ and C_23_ vs pressure for ZnCO_3_-Phase I.

Remarkably, the results found confirm that smithsonite belongs to the group of calcite, since calcite structure is the most stable structure that ZnCO_3_ may adopt at ambient conditions. In order to assess the reliability of our simulation, the mechanical properties of ZnCO_3_ structure-phase I (R-3c space group) are evaluated. The obtained data are fitted to the Birch-Murnaghan equation of state^25^ in order to obtain the bulk modulus, which is in good agreement with the experimental^15^ and previous theoretical results^16^. The set calculated data are listed in Table 4. The dependence of the elastic constants of ZnCO_3_-phase I as a function of pressure variation is calculated from 0 to 90 GPa. Fig. 3 shows that elastic constants increase proportionally with the applied pressure until 78 GPa where a clear discontinuity is noticed, especially for C_11_, C_22_, C_33_, C_12_, C_13_ and C_23_ curves. This continuity supports our prediction regarding the structural transition at this pressure.

**Figure 4:**
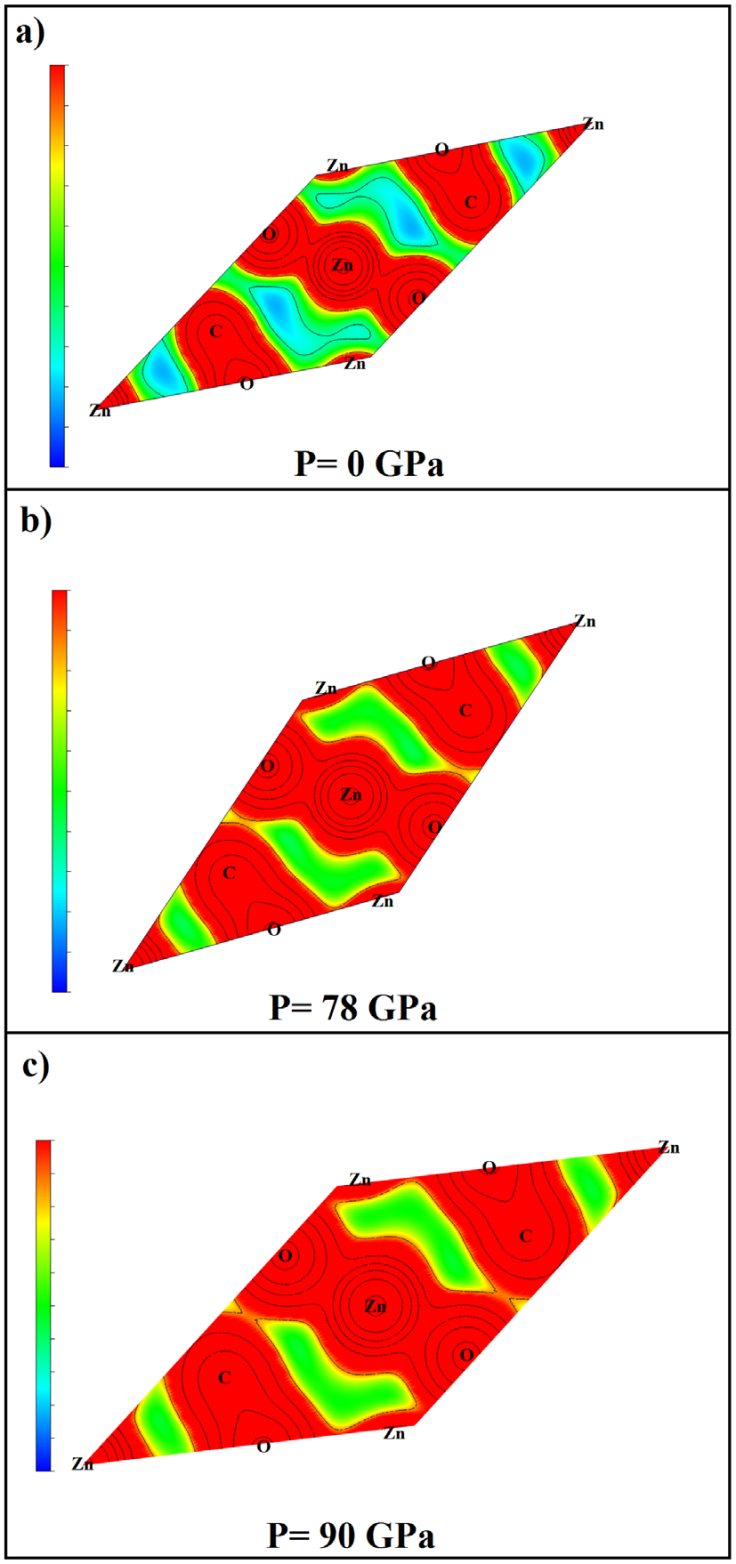
Valence charge density of ZnCO_3_-phase I along the (-3 2 1) plane at zero pressure (a), 78 GPa (b) and 90 GPa (c).

In order to understand the main reasons for the first phase transition, we will analyze - in the following - the electronic charge density. Fig. 4 represents the electronic charge density at 0 GPa (a), 78 GPa (b) and 90 GPa (c) in the (-3 2 1) plane, which includes the three constituents atoms of ZnCO_3_. At zero pressure (Fig. 4.a) there is a maximum of charge transfer between zinc and oxygen and also between oxygen and carbon. Fig. 4.b shows the electronic charge density at 78 GPa, where a maximum of charge is transferred between oxygen and two zinc atoms as well as together between oxygen and carbon-zinc atoms. Consequently, a new bond between oxygen and zinc is formed at that pressure. However at 90 GPa (Fig. 4.c), we notice only a slight new bond between oxygen and carbon.

On the other hand, a Bader charge analysis^26^ of the obtained charge densities is carried out from the present first principles calculations. The charge at the atom is obtained by subtracting the Bader charge from the number of valence electrons considered for that particular atom in the density functional theory (DFT) calculations^27^. The charges at Zn, C and O atoms at different pressures are given in Table 5. Our Bader charge analysis shows that the positive charge at Zn and C decreases by ~0.035*e* and ~0.084*e* respectively, from 1.3839*e* at ambient pressure to 1.3489*e* at 90 GPa for Zn atoms and from 2.1478*e* at ambient pressure to 2.0632*e* at 90 GPa for C atoms. However, the negative charge of O atoms increases by 0.04*e* from −1.1773*e* at ambient pressure to −1.1373*e* at 90 GPa. Therefore, our Bader charge analysis shows a partial electronic charge transfer only from the Zn to O atoms and C to O atoms.

**Figure 5:**
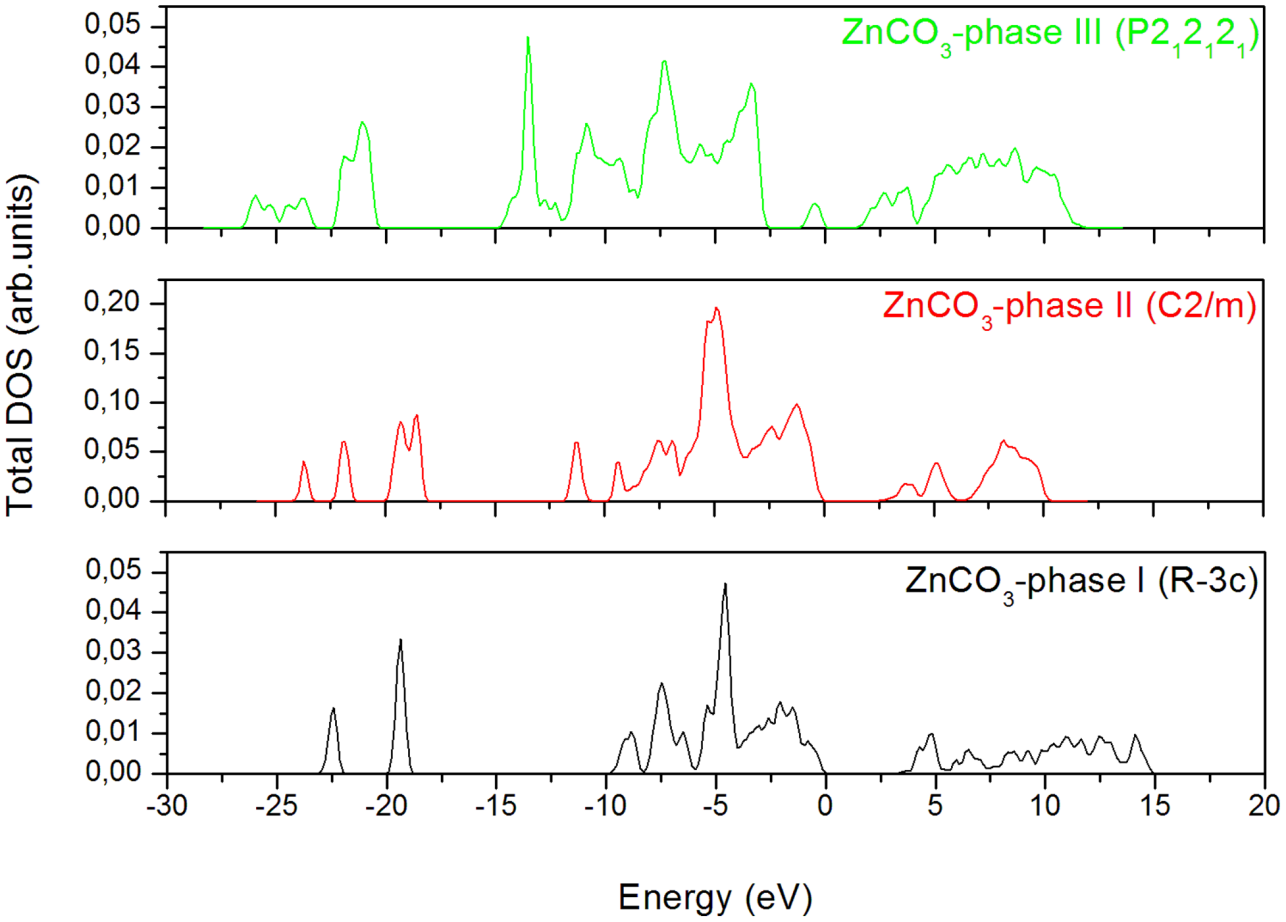
Total density of states (DOS) for each predicted phase of ZnCO_3_. The Fermi level is set to zero.

In order to complete the ground state properties of the obtained phases under pressure, we have – additionally – performed the total density of state for the three phases of ZnCO_3_, as presented in Fig. 5. The obtained band gap value of ZnCO_3_-phase I corresponds to ~3.34 eV, which is slightly higher than the one of the ground state phase II (~2.64 eV), and than the one of the phase III (~1.45 eV). Bouibes et al.^16^ found that the band gap value at the ground state of phase I is around 3.4 eV. The latter is in good agreement with the band gap value of phase I. However, it can be underlined here that the band gap of smithsonite remains closer to some semiconductors, such as ZnO (~3.4 eV), than carbonates such as CaCO_3_ (~6.0 eV), which is rather considered as insulator^7^.

In summary, different phases of ZnCO_3_ were predicted here by means of USPEX method together with DFT. We mainly conclude that below 78 GPa, ZnCO_3_ stable structure has R-3c space group (calcite structure); and between 78 GPa and 121 GPa, ZnCO_3_ takes up a more complex structure (magnesite phase II^7^) with C2/m space group and containing (C_3_O_9_)^6−^ rings of carbonate. Above 121 GPa, the structure of P2_1_2_1_2_1_ space group becomes more stable. The predicted structure is, remarkably, in perfect agreement with the experiment at ambient condition. In addition the computed mechanical quantities at the ground state of phase I agree well with experimental and previous theoretical data. Their behaviors under pressure support our prediction of structural transition of ZnCO_3_ at 78 GPa. Finally, an analysis from electronic charge density and Bader charge was developed to explain the charge transfer that is behind the phase transition.

## Methods

In order to find the stable high-pressure structures of ZnCO_3_, an *ab initio* evolutionary algorithm (EA), as implemented in the “Universal Structure Predictor: Evolutionary Xtallography” (USPEX) code, is employed^19^,^20^,^21^. In this work, the structure prediction runs for ZnCO_3_ were performed at 10 GPa, 20 GPa, 40 GPa, 60 GPa, 90 GPa and 120 GPa, all at zero Kelvin. In these variable-cell simulations, we consider the system with 10, 15 and 20 atoms in the unit cell. The population size is fixed between 20 and 35 number of structures. The first generation is then created randomly. However, in the calculations including 20 atoms/cell, we fix the first generation from the known structures among the other carbonate systems such as calcite, aragonite, post-aragonite, and other structures obtained during the simulations. Magnesite II and magnesite III with 30 atoms in unit cell were added to the resulting structures.

The underlying *ab initio* structure relaxations and enthalpy calculations were carried out using a plane-wave method and the local density approximation (LDA) for the exchange-correlation^22^, as implemented in the Vienna *Ab-initio* Simulation Package (VASP)^23^,^24^. In addition, in order to investigate the fundamental properties of Smithsonite calculations, the exchange and correlation function was treated by means of generalized gradient approximation (GGA). Among the available GGA functional^28^, we selected AM05^29^ functional, which is particularly appropriate for calculating the properties of ZnCO_3_^16^. The electron-ion interaction was described by the all-electron projector augmented wave (PAW) scheme^30^ and the electron configurations 4s^2^, 2s^2^2p^2^ and 2s^2^2p^4^ were treated as valence for Zn, C, and O, respectively. During structural relaxation an energy cutoff of 500 eV was used for the plane wave basis sets, and a k-point resolution of 0.08 Å^−1^ in the reciprocal space was used for all structures in order to minimize the error from the k-point meshes. The atomic positions, lattice parameters, and cell volume were fully relaxed until the force on each atom is less than 1 meV/Å, and stresses deviate from the desired hydrostatic pressure by less than 1 GPa.

## Figures and Tables

**Table 1:** Stable ZnCO_3_ structure (Phase I) between 0 GPa and 78 GPa

*Lattice parameters*				
	a (Å) = 5.71;			
	α(°) = 48.92;			
	V (Å^3^) = 97.21			
*Atomic coordinates (space group R-3c)*				
	Atom	X	Y	Z
	Zn	0.00	0.00	0.00
	C	0.25	0.25	0.25
	O	0.97	0.52	0.25
*Bond length (Å):*				
	Zn--O = 2.14			
	C--O = 1.29			
*Third-order Birch–Murnaghan equation of state (fitted between 0 and 150 GPa)*				
	B_0_ (GPa) = 126.45 (±1.6)			
	B′_0_ = 4.00 (±0)			
	V_0_ (Å^3^) = 98.11(±0.23)			

**Table 2:** Stable ZnCO_3_ structure (Phase II) between 78 GPa and 121 GPa

*Lattice parameters*				
	a (Å) = 9.27;	b (Å) = 7.08;	c (Å) = 8.17	
	β(°) = 104.83;			
	V (Å^3^) = 519.13			
*Atomic coordinates (space group C 2/m)*				
	Atom	X	Y	Z
	Zn1	0.53	0.00	0.19
	Zn2	0.50	0.27	0.50
	Zn3	0.34	0.50	0.14
	C1	0.86	0.83	0.79
	C2	0.74	0.00	0.53
	O1	0.85	0.00	0.45
	O2	0.85	0.00	0.88
	O3	0.60	0.00	0.43
	O4	0.74	0.84	0.64
	O5	0.83	0.68	0.88
	O6	0.99	0.80	0.76
*Bond length (Å):*				
	Zn1--O1 = 2.05; Zn1--O2 = 2.04; Zn1--O5 = 2.22; Zn1--O6 = 2.22			
	Zn2--O1 = 2.16; Zn2--O3 = 2.07; Zn2--O4 = 2.10; Zn2--O6 = 2.04			
	Zn3--O2 = 2.10; Zn3--O6 = 2.05			
	C1--O2 = 1.34; C1--O3 = 1.41; C1--O5 = 1.43; C1--O6 = 1.39			
	C2--O4 = 1.31			
*Third-order Birch–Murnaghan equation of state (fitted between 0 and 150 GPa)*				
	B_0_(GPa) = 180.8(±1.6)			
	B′_0_ = 3.94 (±0.05)			
	V_0_ (Å^3^) = 488.61 (±0.29)			

**Table 3:** Stable ZnCO_3_ structure (Phase III) above 121 GPa

*Lattice parameters*				
	a (Å) = 8.67;	b (Å) = 3.62;	c (Å) = 5.63	
	α(°) = 90.00;	β(°) = 90.00;	γ(°) = 90.00	
	V (Å^3^) = 176.7			
*Atomic coordinates (space group P2_1_2_1_2_1_)*				
	Atom	X	Y	Z
	Zn	0.15	0.66	0.19
	C	0.03	0.54	0.61
	O1	0.05	0.17	0.08
	O2	0.34	0.60	0.02
	O3	0.07	0.80	0.78
*Bond length (Å):*				
	Zn--O1 = 1.9; Zn--O2 = 2.03; Zn--O3 = 2.12			
	C--O1 = 1.35; C--O2 = 1.39; C--O3 = 1.42			
*Third-order Birch–Murnaghan equation of state (fitted between 0 and 150 GPa)*				
	B_0_ (GPa) = 119.15 (±3.2)			
	B′_0_ = 4.36 (±0.07)			
	V_0_ (Å^3^) = 170.92 (±0.33)			

**Table 4:** Mechanical properties of ZnCO_3_ structure (Phase I), at ambient conditions

	Experiment^a^	This work	Theory^b^
C_11_		244.97	243.51
C_22_		240.29	240.24
C_33_		148.79	145.36
C_44_		70.09	69.34
C_55_		44.33	41.31
C_66_		43.25	41.62
C_12_		104.41	103.75
C_13_		73.54	71.13
C_23_		70.49	68.58
B	124	126.45^c^	124.19^c^
		126.86^d^	124.93^d^
B′		4.00	3.99
A		1.00	0.99
G		45.34	44.39
E		121.54	118.98
*ν*		0.34	0.34
V_s_		3.20	3.17
V_p_		6.50	6.43

*a^15^, b^16^, c^25^,d^16^(Voigt formula)*.

**Table 5:** Atomic charge densities (*e*) from Bader charge analysis of Zn, C and O atoms at different pressures

	Atomic charge (*e*) from Bader charge analysis		
Pressure (GPa)	Zn	C	O
0	1.3839	2.1478	−1.1773
50	1.3626	2.1184	−1.1603
70	1.3577	2.0656	−1.1411
78	1.3544	2.0639	−1.1394
90	1.3489	2.0632	−1.1373
